# When Parents Go Shopping: Perspectives on Gender-Typed Toys among Polish Mothers and Fathers from Big Cities

**DOI:** 10.3390/children8090744

**Published:** 2021-08-28

**Authors:** Kornelia Lipowska, Ariadna Beata Łada-Maśko

**Affiliations:** 1 Department of Psychology, Faculty of Social and Behavioural Sciences, University of Amsterdam, 1018 WV Amsterdam, The Netherlands; 2 Department of Developmental Psychology and Psychopathology, Institute of Psychology, Faculty of Social Sciences, University of Gdańsk, 80-309 Gdańsk, Poland; ariadna.lada@ug.edu.pl

**Keywords:** stereotypes, gender, toys, children development, parental attitude

## Abstract

Children learn to understand gender categories and roles typical for women and men from the earliest stages of their life. Toys, by which they are surrounded in everyday routine, may have a significant role in their knowledge of cultural norms and societal expectations, but also might have a great impact on children’s health in the context of psychosocial development. Parental attitudes can also be an important moderator of the choice of gender-stereotyped toys. The aim of the study was to investigate parents’ perspectives on toys’ appropriateness for children according to gender, and to analyze parents’ knowledge about the labeling of toys in shops. In total, 526 parents from big cities participated in the study; to assess parental knowledge and perspectives on toys’ categorization, The World of Toys questionnaire was used. The results indicated that most parents from big cities observe that toys are arranged according to the child’s age and sex and the toys’ functionality. Moreover, in parents’ opinion, the compliance with the role of a woman or man affects categorization the most. Furthermore, both parents tended to attribute toys to both sexes more often than they believe the stores do. To conclude, despite the still visible division of toys in shops, Polish parents from big cities are more liberal and egalitarian than shops are when it comes to the perception of toys’ appropriateness, allowing children to play with most toys regardless of their sex.

## 1. Introduction

The moment they are born, children come into a world with a pre-existing structure and imposed social norms. Gender-normative behaviors are dominant in our culture, including in media and advertisements [1]. Gender stereotypes reflect the differing social roles of women and men [2,3]. These stereotypical beliefs suggest that women are more communal and concerned with others, whereas men are more oriented toward agentic goals [4,5]. Even though approximately half of respondents from Europe think that gender equality has been achieved at work, in politics, and in leadership positions [6], the differing of women and men in social roles is, nonetheless, noticeable in every culture. According to social role theory, gender stereotypes concerning common traits of men and women will not disappear without an equal division of social roles in society [4].

Basic gender attitudes are not inherited—children begin to understand gender categories and their constant placement inside them during development [7]. Additionally, Bem [8] suggests that as a child learns to process information, they develop gender schema—cognitive structures associated with sex-linked characteristics. Early childhood is believed to be crucial for gender socialization—i.e., the construction of sexual identity and gender [9,10]. Therefore, the objects present in toddlers’ everyday lives might relate to their future selves in a significant way. Toys are such objects. One common way in which they are divided is by marketing them to a particular gender. Those so-called gender-typed toys—i.e., toys that are identified as specifically appropriate for either girls or boys in contemporary society [11]—by which children are constantly surrounded might, therefore, play a crucial role in a child’s development.

### 1.1. Characteristics of Gender-Typed Toys

Toys designed for girls and boys are clearly different, and many studies have examined how they vary between genders. Blakemore and Centers [12] examined, with the use of the Internet, catalogues, and advertisements for children, which kinds of toys are recognized as being designed for a particular gender, thereby identifying representative groups. They found that toys can be categorized as strongly feminine, slightly feminine, neutral, slightly masculine, and strongly masculine. The study showed that toys perceived as being mostly for girls were often associated with appearance, while those seen as being for boys were correlated with violence and aggression. Additionally, men’s ratings were slightly more gender-stereotyped than women’s ratings.

Although Blakemore and Centers did not focus on this aspect [12], it was suggested by Miller [13] that play objects for girls are associated with domestic life, while play objects for boys encourage more symbolic play, often removed from daily life. Furthermore, girls’ toys appear to be more related to manipulability, nurturing, and creativity, whereas boys’ toys were rated much more highly on constructiveness, competitiveness, and, as previously mentioned, aggressiveness.

These characteristics of gender-typed toys were examined more widely in a further study carried out by Reich, Black, and Foliaki [14]. The authors contrasted two widely known categories of LEGO^®^: LEGO^®^ City^®^, which is marketed to boys, and LEGO^®^ Friends^®^, which is marketed to girls. The thematic analysis acknowledged that both of the LEGO^®^ categories set gendered narratives, particularly through the stereotypical roles of the LEGO^®^ characters. First of all, the sets of thematic patterns that emerged from the data showed that male characters had professional roles, whereas the recreational activities and domestic tasks of the female characters were emphasized. Furthermore, the LEGO^®^ City^®^ characters were associated with dangerous actions and saving people—firefighters, for example—while the LEGO^®^ Friends^®^ characters were associated with socializing and caring for each other—for example, a focus on interaction with other Friends^®^ characters. The final thematic pattern was that the narratives of female characters were often associated with beauty and attractiveness. These gendered narratives illustrate how girls’ and boys’ play patterns can vary enormously [14].

It should be noted that this study did not involve children, so it could not show whether these gendered narratives are perceived by children. In addition, one must recall that LEGO^®^, no matter which category, can be very educationally beneficial [15]. However, the content analysis showed that the set narratives are highly gendered in how they show children how to play with them.

The presented studies illustrate that from the beginning of their development, children are exposed to subtle gender-related messages [9]. Already in early childhood, toy objects suggest to toddlers that the social role of men is associated with heroism, danger, aggression, and violence, whereas the role of women involves socializing and caring about others and one’s own appearance. Moreover, children are shown that men have professions, whereas women have hobbies. This striking division demonstrates that toys—objects that play an important role in children’s daily lives—are highly gendered.

Thus, from very early in their development, children are taught about associations related to gender and the dichotomy between males and females present in many aspects of life [8]. Toys can shape how children engage with and what they learn from the activities that they undertake [14]. Through play, children can gain knowledge of cultural norms and societal expectations, as play allows them to observe and practice adults’ social roles [16]. Therefore, such exposure to stereotypically designed play objects might result in future gender differences.

### 1.2. Moderator of Choosing Gender-Typed Toys

Knowing the moderators of children’s choice of gender-stereotyped toys may make it easier to understand what can be done to minimize the consequences of gender-typed play patterns and the stereotypical division between the sexes. In spite of the decrease in social gender restrictions, gendered play patterns still exist [17]. The most important origin of gender differences in children’s toy preferences is in social factors, considering cognitive development—i.e., age-related approaches to skills and translating thoughts into actions [18]. According to Kohlberg’s cognitive developmental theory [7], self-socialization is the most important aspect of the process of gender role development. Children look for information in the environment about gender roles in order to recognize their own gender group and to behave like other members of this group. Gender schema theory [8] solidifies Kohlberg’s idea, as it proposes that it is natural to look for information associated with sex to consolidate the perception of gender and to relate to it different skills, including psychosocial skills.

Due to the positive reinforcement associated with seeing that doing something is rewarded [7], parental attitudes toward the selection of toys are also an important factor in a child’s psychosocial development related to gender differences. Furthermore, parents are the ones providing toddlers with toys, and therefore sending gendered messages about the appropriateness of the toys. A study by Kollmayer, Schultes, Schober, Hodosi, and Spiel [19] aimed to better understand the role of parents in encouraging children’s stereotypically gendered play by examining various aspects of parents’ attitudes toward gender roles and gender-typed toys. The study revealed that parents rated gender-neutral and same-gender-typed toys as equally more desirable for their children than cross-gender-typed toys. Moreover, traditional gender role attitudes were found to be correlated with rejecting the cross-gender-typed toys, but there was no relationship with attitudes toward same-gender-typed toys. Parents with traditional views of gender roles judged more positively the gender-typing of toys than parents with an egalitarian point of view did.

This shows that parental attitude can be an important moderator of the choice of gender-stereotyped toys. However, contrary to what Kollmayer et al. [19] indicated, there is plenty of research indicating the importance of sex bias toward playing with gender-typed toys. Nowadays, toys stereotypically marked as feminine are rated as more gender-typed than stereotypically masculine toys [20]. This leads to boys being discouraged from playing with feminine toys, which, in turn, leads them to avoid doing so. In contrast, girls are not discouraged from playing with cross-gender-typed toys [21], and they even often choose them over feminine toys. Therefore, it is possible that stereotypical psychosocial effects will endure longer for boys.

One way that parents and children identify gender-appropriate toys is through labels defining them as “for girls” or “for boys” in shops, rather than “for children” in general [11]. Moreover, toys are not only labeled verbally, but also through implicit gender-typed color labels—pink and blue [22].

As indicated, shops are the main suppliers of toys, and parents are most often the ones that provide them directly to children. Therefore, the aim of the study was to investigate parents’ perspectives on toys’ appropriateness for children according to gender, and also to analyze parents’ knowledge about the labeling of toys in shops. In spite of much evidence about the gender narratives presented by toys and parental attitude as a moderator of play objects, there is still no clear link between parents’ point of view regarding toys’ appropriateness according to children’s gender and the labels provided by shops.

Furthermore, in recent years, the involvement of fathers in children’s upbringing has greatly increased [23], so it is important to investigate the difference between the attitudes of mothers and fathers regarding toys. Moreover, as Poland is known to be a very conservative country [24], we decided to focus our research on the attitudes of Poles. With this, we want to obtain a better perspective on the future of children living in such traditional countries and to see if there is hope for a society with less gender-based discrimination and exclusion and, therefore, a better world for future generations, regardless of sex and gender.

Thus, we created three research questions:

1. What is parents’ awareness of the labeling of toys in shops?
2. How do parents perceive toys’ appropriateness according to children’s gender? Does parents’ gender differentiate their perspective?
3. Does parents’ awareness of the labeling of toys in shops differ from their own perspective on toys’ appropriateness according to children’s gender?

## 2. Materials and Methods

### 2.1. Participants and Procedure

Participants were selected through selective sampling. Only parents with Polish nationality from Tricity were invited to the study. Tricity is a metropolis in northern Poland that consists of three cities: Gdańsk, Gdynia, and Sopot. Considering its population structure according to demographic and economic criteria, Tricity might be recognized as a good example of a large city in Poland [25]. Due to special circumstances related to the COVID-19 pandemic, the study was conducted via an online questionnaire and participants were invited by e-mail and with the use of social media platforms. Moreover, people who responded were asked to invite their colleagues who also met the inclusion criteria [22]; i.e., the non-random sampling method known as “snowball sampling”. This study used data collected in January 2021, just after the Christmas holiday, during which there is the greatest number of purchases of toys and visits in toy shops recorded thorough the year in Poland [26]. At the beginning of the questionnaire, the goal of the study was explained, and people were asked to give their consent and were informed that participation was anonymous and voluntary. As a result of the short time, precisely one month, in which data were collected, 653 responses were received. However, due to the errors of some respondents in filling out the questionnaire, the final sample totaled *N* = 526, comprising 376 mothers and 150 fathers. The average age of the mothers was *M* = 36.01 (*SD* = 6.13), and of the fathers, *M* = 40.78 (*SD* = 6.99). Furthermore, information about parental education levels (0.6% had primary education, 1.5% vocational education, 11.4% secondary education, and 86.5% higher education) and the number of children (36.5% had one child, 47.3% two children, 13.3% three children, 1.9% four children, and 0.8% had five children) was collected. Among all parents, 31.4% had only daughters, 29.5% only had sons, and 39.2% had children of both sexes.

### 2.2. Methods

For the purpose of this study, a questionnaire entitled “The World of Toys” was developed by Lipowska and Łada-Maśko (see Appendix A). The aim of the questions was to assess parental knowledge and perspectives on toys’ categorization. A pilot study was conducted on a group of 30 parents. The final version of the questionnaire consisted of 10 questions, divided into three parts: general information about the parent, their awareness of toys’ labeling in shops, and parents’ own perspectives on toys’ appropriateness according to children’s gender. After responding to the first part, parents were asked to choose the categories in which they believed shops label toys. Then, they categorized the presented groups of toys as either for boys, girls, or both according to their opinion on the labeling of toys in shops, as well as their own perspective of toys’ appropriateness according to children’s gender. The list of groups of toys to classify was provided by competent judges. The questionnaire’s validity, assessed using Cronbach’s α, was 0.84.

## 3. Results

Statistical analyses were carried out using the Statistical Package for the Social Sciences (SPSS) version 26 (SPSS Inc.; license purchased by University of Gdańsk). The chi-squared statistic tests were used in order to analyze differences between studied variables.

### 3.1. Parents’ Awareness of Toys’ Labeling in Shops

In order to answer the first research question regarding parents’ awareness of the labeling of toys in shops, parents’ responses to questions number 6 (“Did you notice that toys in stores are arranged according to some criteria?”), 7 (“In most stores, toys are arranged according to certain categories. What are these categories in your opinion?”), and 9 (“What characteristics of the toy, in your opinion, affect its categorization for girls or for boys?”) in The World of Toys questionnaire were analyzed.

Overall, 90.7% of parents declared that they noticed that toys in stores were arranged according to some criteria, while 9.3% of parents did not notice that at all. Furthermore, in parents’ opinions, toys are arranged according to certain categories, as shown in Table 1.

Mothers’ and fathers’ differences were statistically significant in their estimates of arranging the toys according to functionality, the child’s sex, and the company. In all three cases, the mother’s mentioned the category more often than the fathers (see Table 1). However, mothers chose the fabric of the toy more often than its price as a category of arranging toys, while fathers chose the opposite (see Table 2 and Table 3).

The results also suggested that parents’ differences were not statistically significant in their estimates of characteristic of toys affecting their categorization for girls or for boys (Table 4). However, the results indicated that mothers chose the compliance with the role of a woman or man significantly more often than appearance, and color more often than function and appearance (see Table 5). In case of fathers, there were no statistically significant differences (see Table 6). Moreover, mothers chose the color of the toy significantly more often than its function as a trait influencing the categorization of toys (see Table 5), while fathers chose the opposite (see Table 6), but in case of fathers, the differences were not statistically significant.

### 3.2. Parents’ Perception of Toys’ Appropriateness According to Children’s Gender

Subsequently, in order to answer the second research question, parents’ answers to question number 10 (“According to you, which of the following toys are typical toys for boys and which are typically toys for girls?”) from The World of Toys questionnaire were analyzed. Here, parents rated toys as typical for girls, typical for boys, or the same for both sexes (see Table 7).

The results indicated that most of the toys assessed by mothers and fathers were intended for both sexes; however, mothers were more likely than fathers to judge toys as intended for children of both sexes—especially those toys culturally assigned to the female role; e.g., cooker or baby dolls, or the male role; e.g., vehicles or military toys—than as toys typical for girls or as typical for boys.

The results also showed that mothers and fathers significantly differed from each other in their assessment of the toys’ appropriateness according to children’s gender—only creative toys were assessed similarly by both mothers and fathers. Furthermore, the greatest effect sizes of the above differences were observed in the cases of cooker, baby dolls, tools, and vehicles—average effect size (see Table 7), which also confirmed that parents differ the most in case of toys culturally assigned to the female or male role.

### 3.3. Differences between Parents’ Awareness of Labeling of Toys in Shops and Their Own Perspective on Toys’ Appropriateness According to Children’s Gender

Lastly, in order to answer the third research question, parents’ answers to questions number 8 (“Which of the following toys do you think SHOPS categorize as toys typically for boys, and which are typically toys for girls?”) and 10 (“According to YOU, which of the following toys are typical toys for boys and which are typically toys for girls?”) from The World of Toys questionnaire were analyzed. Parents rated the toys as typical for girls, typical for boys, or the same for both sexes, and also assessed the labeling of them by shops in the parents’ opinion. The results indicated that both mothers and fathers tended to attribute toys to both sexes more often than they believed stores do, which was particularly evident in the case of toys stereotypically attributed to male or female roles, such as baby dolls, cooker, vehicles, and military toys (see Table 8 and Table 9). Again, only in the case of a toy cooker was there no statistically significant difference between fathers’ perspectives on the toy’s appropriateness for a child’s gender and the labeling of the toy in shops in the fathers’ opinion. Average and large effect sizes were mostly observed, which confirmed that mothers and fathers tended to attribute toys different than they believe stores do.

## 4. Discussion

In this study, we investigated parental awareness of the categorization of toys in shops, especially in regard to children’s gender, parents’ own perspectives on toys’ appropriateness according to children’s gender, and the difference between those two.

Our results revealed that Polish parents living in big cities are aware of the labeling of toys in shops, and that the overarching categorization criterion is the compliance with gender roles. In Poland, those roles of men and women are greatly emphasized [27]. This gender inequality is present not only in workplace settings, but also in family and public spheres, which might greatly restrict the opportunities in life of women in particular, as Poland is a country with patriarchal structure defined by the Catholic Church in which men have higher social status than women [28]. For example, recently, a new law in Poland was enforced that imposed a near-total ban on abortion, only allowing abortion in case of rape, incest, or danger to the mother’s life [29]. The termination of pregnancy was banned even in cases of severe and fetal abnormalities, making it one of the strictest abortion laws in Europe [30] and violating women’s fundamental human rights [31]. However, it should be noted that also not all men have social privileges. The men in Poland who have the highest social status are white, educated, employed, able to serve in the military, heterosexual, Catholic, and married with children [32]. The pressure among men to conform to this ideal of masculinity and among those who are never going to achieve this standard (for instance, sexual and gender minorities) might cause them to experience great stress. The suicide ratio of Polish men is 7.4 times higher compared to women, and the overall ratio of suicide occurrence is one of the highest in the entire world [33]. Therefore, such stereotypical beliefs of men and women and the gender biases resulting from them might be further consolidated and enhanced by the visible categorization of toys in shops according to traditional gender roles, promoting children’s specific and limited visions of future professions. Furthermore, providing categorizing labels such as “for girls” and “for boys” prematurely sets boundaries between the children to whom the toy is and is not marketed; hence, it can lead to the exclusion of one gender [34], which might persist into adulthood. This phenomenon of social exclusion erects barriers to education for both girls and boys. Gender differences in certain fields and skills are called gender gaps [35]. Despite the fact that boys were historically given more opportunities in the field of learning [36], they often underachieve relative to girls in many developed countries. In spite of their frequent advantage in mathematics, they often perform worse in terms of reading abilities [37]. Moreover, this gender gap in reading is three times larger than that in math. Reinforcement of this negative stereotype is influenced by cognitive processes and might lead to lower achievements [38]; therefore, one might deduce that toys, which are important in cognitive development, can play a crucial role. Play objects associated with aggression and loud play patterns (targeted at boys) may affect children’s capacity to remain silent and concentrated at early stages of education [39]. Although boys underperform during childhood in school, in adulthood, men enjoy a variety of advantages [40]. Women are often excluded from education, even though female education has a profound impact on a society in terms of lower fertility, reduced infant and child mortality, and children’s upbringing [41]. Moreover, the targeting of toys to specific genders leads to different play patterns, which might have an impact on children’s future artistic and motor skills [12]. Many toys targeted at boys, to the exclusion of girls, are associated with construction; e.g., blocks or puzzles, and are correlated with spatial and mathematical skills [42]. Girls’ toys, like dolls or domestic items, are rated higher on manipulability [13]. This distribution might lead to lower levels of certain skills among both sexes.

However, it is noteworthy that although parents are aware of the gender-normative labeling of toys in shops, our research revealed that their own attitudes do not always comply, especially in the case of mothers. Both parents judged most toys as appropriate for both sexes, but mothers’ views were even more egalitarian in regard to those toys that are culturally assigned to one of the sexes. Despite the growing recognition of the positive effects of equal distribution of household labor, women are still the ones who undertake most activities regarding children’s care [3]—for instance, providing children with toys. This implies that gender roles may become more flexible, contributing to greater diversity of future generations.

Additionally, mothers chose the color of the toy as the most influencing trait of categorization according to gender appropriateness. This confirms that toys are not only labeled verbally, but also through implicit gender-typed color labels—pink and blue [22]. Moreover, this finding also provides a way of expanding children’s spectrum of accessible toys without deeming them inappropriate according to gender, namely by using counter-stereotypical colors. For example, the LEGO^®^ Corporation has a collection with pink bricks to match girls’ preferences and identification [43].

Interestingly, our results suggest that children’s sex did not influence parents’ perception of toys’ appropriateness, as toys stereotypically assigned to one sex, such as a toy cooker or tools, were also perceived as such by both parents, especially by fathers, whose views were less egalitarian than those of mothers. Therefore, although a child’s sex can often moderate various parental attitudes [9], it is not related to their perception of toys’ appropriateness. As already mentioned, only the sex of parents was a significant moderator of the perception of a toy’s appropriateness, as fathers judged play objects more conservatively than mothers did. The only toy that was seen by a great majority of both parents as only for girls and by almost no one as for boys was Barbie dolls. This result is not surprising, as the Barbie brand advertises their dolls exclusively to girls [44], not only through verbal statements, but also through the usage of the color pink, socially perceived as a gender label [22]. However, it is important to state that playing with Barbie dolls might be damaging to girls’ body image [45] by showing unrealistically thin body ideals, and might also exaggerate gendered perceptions of women by the expression of the hyper-femininity of those dolls [46]. Therefore, it might be advisable that even girls should play less with Barbie dolls. However, on the positive side, the Dream Gap Project [47] is an attempt by the Barbie brand to help girls to realize their limitless potential and possibilities, thereby closing the Dream Gap (limiting self-beliefs developed by the age of 5). This global initiative highlights positive role models, funds research, and produces inspiring products and content. For instance, new astrophysicist and astronaut Barbie models have been released, motivating girls to pursue stereotypically male jobs [48]. However, it should still be noted that such a project is an attempt to allow girls into the “world of boys”, expanding their opportunities, but not the other way around—boys are still excluded from this part of the “girls’ world”. Not all boys may want to become a firefighter or soldier, as the toys aimed at them suggest [14], but they might also want work in professions stereotypically considered feminine; for instance, as nurses or childcare workers [49]. Therefore, it is possible that some stereotypical play patterns will persist longer for boys.

## 5. Conclusions

The main conclusion of our study is that despite the still-visible division of toys in shops according to children’s sex, the majority of Polish parents from big cities are more liberal and egalitarian than shops are when it comes to perceptions of toys’ appropriateness, allowing their children to play with most play objects, regardless of their sex. This is especially true for mothers, which is an uplifting finding, as mothers are still often the primary caretakers of children [3], and their allowance of play with counter-stereotypical toys might be a factor contributing to the creation of a society with less gender-based discrimination. However, it is important to note that the presentation of toys in shops is addressed not only to people from big cities, but also to those from small cities and villages that often have more traditional views regarding roles of men and women [50]. Therefore, as the shop offer must be appealing to all groups of customers, it is not surprising that the stereotypical division of toys in shops is still present.

## 6. Limitations and Future Research

Several limitations of the current study need to be addressed while considering the results. One of the main limitations of our research was its explorative character, which did not allow for deriving conclusions about cause–effect associations. Moreover, the declarative nature of the collected data was another important limitation, which should be addressed in future studies by directly investigating parents’ behaviors in real-life situations—for instance, in toy shops. The results must also be interpreted carefully because of the lack of inclusion of the age of the children and family factors (e.g., family size, opposite- or same-sex parents) in the study. Further research including an inter-relationship analysis between different familial factors and their influence on parental attitudes regarding toys’ appropriateness is required. It would be also interesting to investigate how psychological factors (e.g., personality factors, parental sexism, etc.) might influence parental attitudes. Moreover, in this study, only Polish parents from big cities were examined, thus limiting the generalizability of the results. In future research, it would be extremely valuable to involve parents from different places of residence, especially villages and small cities, as well as other countries, to represent divergent cultures.

## Figures and Tables

**Table 1:** Categories of arranging toys in stores—differences in parents’ opinions.

Category of Arranging Toys	% of Mothers	% of Fathers	*χ* ^2^	*df*	*p*	*Phi*
Child age	88.8	83.3	2.91	1	0.088	0.07
Functionality	76.6	63.3	9.53	1	**0.002**	0.14
Child sex	72.6	63.3	4.39	1	**0.036**	0.09
Company	70.5	61.3	4.11	1	**0.043**	0.08
Fabric	16.2	10.7	2.65	1	0.104	0.07
Price	8.5	11.3	1.01	1	0.315	0.04

*Note*. Bold type indicates significant differences.

**Table 2:** Categories of arranging toys in stores—differences in mothers’ opinions.

	*χ* ^2^ *(p) Phi*					
Category of Arranging Toys by Mothers	1	2	3	4	5	6
1. Child age	X					
2. Functionality	5.69 **(0.017)** 0.12	X				
3. Child sex	32.35 **(<0.001)** 0.29	1.13 (0.288) 0.05	X			
4. Company	4.04 **(0.044)** 0.10	0.65 (0.420) 0.04	7.21 **(0.007)** 0.14	X		
5. Fabric	6.66 **(0.010)** 0.13	11.53 **(0.001)** 0.18	11.29 **(0.001)** 0.17	18.45 **(<0.001)** 0.22	X	
6. Price	18.98 **(<0.001)** 0.23	1.20 (0.273) 0.06	3.08 (0.079) 0.09	1.95 (0.163) 0.07	11.65 **(0.001)** 0.18	X

*Note*. Bold type indicates significant differences; *df* = 1.

**Table 3:** Categories of arranging toys in stores—differences in fathers’ opinions.

	*χ* ^2^ *(p) Phi*					
Category of Arranging Toys by Fathers	1	2	3	4	5	6
1. Child age	X					
2. Functionality	1.66 (0.198) 0.11	X				
3. Child sex	28.94 **(<0.001)** 0.44	4.21 **(0.040)** 0.17	X			
4. Company	3.80 (0.051) 0.16	0.364 (0.546) 0.05	2.71 (0.100) 0.13	X		
5. Fabric	3.58 (0.058) 0.16	7.14 **(0.008)** 0.22	4.50 **(0.034)** 0.17	0.42 (0.519) 0.05	X	
6. Price	4.79 **(0.029)** 0.18	2.19 (0.139) 0.12	0.89 (0.345) 0.08	0.05 (0.821) 0.02	7.07 **(0.008)** 0.22	X

*Note.* Bold type indicates significant differences; df = 1.

**Table 4:** Characteristics of toys affecting their categorization for girls or for boys—differences in parents’ opinions.

Characteristic of Toys	% of Mothers	% of Fathers	*χ* ^2^	*df*	*p*	*Phi*
Compliance with the role of a woman or man	78.7	78.7	0.00	1	0.989	0.00
Color	71	66.7	0.96	1	0.327	0.04
Function	62.5	68.7	1.78	1	0.183	0.06
Appearance	38.8	42.7	0.66	1	0.417	0.04

**Table 5:** Characteristics of toys affecting their categorization for girls or for boys—differences in mothers’ opinions.

		*χ* ^2^ *(p) Phi*		
Characteristic of Toys by Mothers	1	2	3	4
1. Compliance with the role of a woman or man	X			
2. Color	0.25 (0.615) 0.03	X		
3. Function	0.61 (0.435) 0.04	10.61 **(0.001)** 0.17	X	
4. Appearance	4.21 **(0.040)** 0.11	20.31 **(<0.001)** 0.23	1.32 (0.251) 0.06	X

*Note.* Bold type indicates significant differences; *df* = 1.

**Table 6:** Characteristics of toys affecting their categorization for girls or for boys—differences in fathers’ opinions.

		*χ* ^2^ *(p) Phi*		
Characteristic of Toys by Fathers	1	2	3	4
1. Compliance with the role of a woman or man	X			
2. Color	1.27 (0.260) 0.09	X		
3. Function	1.63 (0.201) 0.10	3.04 (0.081) 0.14	X	
4. Appearance	0.07 (0.792) 0.02	1.36 (0.243) 0.10	0.11 (0.736) 0.003	X

**Table 7:** Perception of toys’ appropriateness according to children’s gender—parents’ opinions.

Toys	% of Mothers			% of Fathers			Differences			
	For Girls	For Boys	For Both Sexes	For Girls	For Boys	For Both Sexes	*χ* ^2^	*df*	*p*	*Cramer’s V*
Figures of animals and characters	1.1	3.2	**95.7**	7.3	6.7	**86**	18.97	2	<0.001	0.19
Computer games	0	14.1	**85.9**	0.7	37.3	**62**	38.19	2	<0.001	0.27
Board games	1.3	0.8	**97.9**	1.3	6.7	**92**	15.33	2	<0.001	0.17
Musical instruments	2.1	0.8	**97.1**	9.3	2	**88.7**	15.47	2	<0.001	0.17
Blocks	0	4.8	**95.2**	0.7	16	**83.3**	21.04	2	<0.001	0.20
Cooker	31.1	0	**68.9**	**68**	0.7	31.3	63.53	2	<0.001	*0.35*
Baby dolls	**55.6**	0	44.4	**88**	0.7	11.3	53.43	2	<0.001	*0.32*
Adventure dolls	8.8	31.9	**59.3**	15.3	**48.7**	36	23.59	2	<0.001	0.21
Barbie dolls	**70.2**	0.3	29.5	**90**	0	10	22.99	2	<0.001	0.21
Tools	0.5	38.6	**60.9**	0.7	**71.3**	28	46.60	2	<0.001	*0.30*
Plush toys	3.5	0.3	**96.3**	10	1.3	**88.7**	11.42	2	0.003	0.15
Vehicles	0	33.5	**66.5**	1.3	**62.7**	36	44.05	2	<0.001	*0.30*
Puzzle	0.8	1.1	**98.1**	4	2	**94**	7.32	2	0.026	0.12
Artistic toys	4.3	0.5	**95.2**	10.7	0.7	**88.7**	7.77	2	0.021	0.12
Creative toys	3.2	0.8	**96**	5.3	2.7	**92**	4.29	2	0.117	0.09
Military toys	0.3	**54.5**	45.2	1.3	**82.7**	16	40.53	2	<0.001	0.28
Sports toys	0	5.3	**94.7**	1.3	16.7	**82**	23.05	2	<0.001	0.21

*Note.* Bold type indicates the parent’s most frequently chosen answer; italics indicates the greatest effect sizes.

**Table 8:** Differences between mothers’ perspectives on toys’ appropriateness according to children’s gender and their awareness of labeling of toys in shops according to children’s gender.

Toys	Mothers’ Perspective			Labeling of Toys in Shops—Mothers’ Opinion			Differences			
	For Girls	For Boys	For Both Sexes	For Girls	For Boys	For Both Sexes	*χ^2^*	*df*	*p*	*Cramer’s V*
Figures of animals and characters	1.1	3.2	**95.7**	6.6	9.3	**84**	75.59	4	<0.001	*0.32*
Computer games	0	14.1	**85.9**	0.8	46.8	**52.4**	45.76	2	<0.001	*0.35*
Board games	1.3	0.8	**97.9**	2.4	2.4	**95.2**	106.08	4	<0.001	*0.38*
Musical instruments	2.1	0.8	**97.1**	4.8	2.1	**93.1**	55.72	4	<0.001	0.27
Blocks	0	4.8	**95.2**	1.3	22.3	**76.3**	27.15	2	<0.001	0.27
Cooker	31.1	0	**68.9**	**85.4**	0.5	14.1	13.75	2	0.001	0.19
Baby dolls	**55.6**	0	44.4	**97.1**	0.5	2.4	7.42	2	0.024	0.14
Adventure dolls	8.8	31.9	**59.3**	11.4	**67.6**	21	239.32	4	<0.001	*0.56*
Barbie dolls	**70.2**	0.3	29.5	**97.9**	0.5	1.6	4.48	4	0.045	0.08
Tools	0.5	38.6	**60.9**	1.3	**91.2**	7.4	17.98	4	0.001	0.16
Plush toys	3.5	0.3	**96.3**	13.3	0.3	**86.4**	19.34	4	0.001	0.16
Vehicles	0	33.5	**66.5**	1.1	**87**	12	19.67	2	<0.001	0.23
Puzzle	0.8	1.1	**98.1**	2.4	1.3	**96.3**	85.39	4	<0.001	*0.34*
Artistic toys	4.3	0.5	**95.2**	16.2	1.1	**82.7**	42.80	4	<0.001	0.24
Creative toys	3.2	0.8	**96**	17.6	3.5	**79**	46.26	4	<0.001	0.25
Military toys	0.3	**54.5**	45.2	2.1	**93.4**	4.5	19.80	4	0.001	0.16
Sports toys	0	5.3	**94.7**	0.8	34	**65.2**	34.96	2	<0.001	0.31

*Note.* Bold type indicates the parents’ most frequently chosen answer; italics indicates the greatest effect sizes.

**Table 9:** Differences between fathers’ perspectives on toys’ appropriateness according to children’s gender and their awareness of labeling of toys in shops according to children’s gender.

Toys	Fathers’ Perspective			Labeling of Toys in Shops—Fathers’ Opinion			Differences			
	For Girls	For Boys	For Both Sexes	For Girls	For Boys	For Both Sexes	*χ* ^2^	*df*	*p*	*Cramer’s V*
Figures of animals and characters	7.3	6.7	**86**	10	10	**80**	86.48	4	<0.0001	*0.54*
Computer games	0.7	37.3	**62**	1.3	**59.3**	39.3	46.27	4	<0.001	*0.39*
Board games	1.3	6.7	**92**	3.3	10.7	**86**	74.33	4	<0.001	*0.50*
Musical instruments	9.3	2	**88.7**	9.3	7.3	**83.3**	61.05	4	<0.001	*0.45*
Blocks	0.7	16	**83.3**	3.3	33.3	**63.3**	52.18	4	<0.001	*0.42*
Cooker	**68**	0.7	31.3	**92.7**	0.7	6.7	4.53	4	0.339	0.12
Baby dolls	**88**	0.7	11.3	**98**	0.7	1.3	15.97	4	0.003	0.23
Adventure dolls	15.3	**48.7**	36	19.3	**59.3**	21.3	94.36	4	<0.001	*0.56*
Barbie dolls	**90**	0	10	**96**	2	2	11.19	2	0.004	0.27
Tools	0.7	**71.3**	28	4	**90.7**	5.3	33.43	4	<0.001	*0.33*
Plush toys	10	1.3	**88.7**	14	1.3	**84.7**	85.08	4	<0.001	*0.53*
Vehicles	1.3	**62.7**	36	2.7	**85.3**	12	22.97	4	<0.001	0.28
Puzzle	4	2	**94**	3.3	8	**88.7**	75.53	4	<0.001	*0.50*
Artistic toys	10.7	0.7	**88.7**	16	3.3	**80.7**	59.83	4	<0.001	*0.45*
Creative toys	5.3	2.7	**92**	6	7.3	**86.7**	61.99	4	<0.001	*0.46*
Military toys	1.3	**82.7**	16	2.7	**94.7**	2.7	11.36	4	0.023	0.20
Sports toys	1.3	16.7	**82**	5.3	32	**62.7**	76.65	4	<0.001	*0.51*

*Note.* Bold type indicates the parents’ most frequently chosen answer; italics indicates the greatest effect sizes.

## Data Availability

The data presented in this study are available upon request from the corresponding author.

## Appendix A

     *The World of Toys*

Dear Sir or Madam,

We cordially invite you to take part in a short study on toys that you see in stores. The questionnaire was developed by researchers from the Faculty of Psychology at the University of Amsterdam and the Institute of Psychology at the University of Gdańsk.

We kindly ask you to answer a few following questions. Please remember that there are no right or wrong answers and usually the first thought is the most accurate.

The participation in the research is voluntary and the collected data is anonymous, for research purposes only. You can refuse to participate in the study at any time.

The duration of the test is approximately 10 min.

1. You are:
  □ Mother
  □ Father
2. Please provide your age (years): …………………………….
3. What is your education level?
  □ Primary education
  □ Vocational education
  □ Secondary education
  □ Higher education
4. Place of residence (please enter the name): …………………
5. Select the sex and age of your child /children (using X):

	Girl	Boy	0–2 Years Old	3–5 Years Old	6–9 Years Old	10–15 Years Old	16–18 Years Old	Above 18 Years Old
First child								
Second child								
Third child								
Fourth child								
Fifth child								

6. Did you notice that toys in stores are arranged according to some criteria?
  □ Yes
  □ No
7. In most stores, toys are arranged according to certain categories. What are these categories in your opinion? You can choose more than one answer.
  □ Child’s age (e.g., infant, 2–5 years old)
  □ Child’s sex (for boys, for girls)
  □ Company (e.g., LEGO^®^, Fisher Price^®^, etc.)
  □ Functionality (e.g., educational toys, games, blocks, etc.)
  □ Fabric (e.g., wood, plastic)
  □ Price
  □ Other: ………………..
8. Which of the following toys do you think SHOPS categorize as toys typically for boys, and which are typically toys for girls? Mark the answer on each line—using X.

**Toys**	**For Girls**	**For Boys**	**For Both Sexes**
Figures of animals and characters			
Computer games			
Board games			
Musical instruments			
Blocks			
Cooker			
Baby dolls			
Adventure dolls			
Barbie dolls			
Tools			
Plush toys			
Vehicles			
Puzzle			
Artistic toys			
Creative toys			
Military toys			
Sports toys			

9. What characteristics of the toy, in your opinion, affect its categorization for girls or for boys? You can choose more than one answer.
  □ Colour
  □ Appearance
  □ Function
  □ Compliance with the role of a woman/man
  □ Other: ………………
10. According to YOU, which of the following toys are typical toys for boys and which are typically toys for girls? Mark the answer on each line—using X.

**Toys**	**For Girls**	**For Boys**	**For Both Sexes**
Figures of animals and characters			
Computer games			
Board games			
Musical instruments			
Blocks			
Cooker			
Baby dolls			
Adventure dolls			
Barbie dolls			
Tools			
Plush toys			
Vehicles			
Puzzle			
Artistic toys			
Creative toys			
Military toys			
Sports toys			

     Thank you for taking part in the study

## References

[1] Tartaglia S., Rollero C. (2015). Gender Stereotyping in Newspaper Advertisements: A Cross-Cultural Study. J. Cross. Cult. Psychol..

[2] Eagly A.H., Karau S.J. (2002). Role congruity theory of prejudice toward female leaders. Psychol. Rev..

[3] Kosakowska-Berezecka N., Korzeniewska L., Kaczorowska M. (2016). Sharing housework can be healthy: Cultural and psychological factors influencing men’s involvement in household maintenance. Health Psychol. Rep..

[4] Eagly A.H., Wood W., Van Lange P.A.M., Kruglanski A.W., Higgins E.T. (2012). Social role theory. Handbook of Theories of Social Psychology.

[5] Rollero C., Tartaglia S. (2013). Men and women at work: The effects of objectification on competence, pay, and fit for the job. Stud. Psychol..

[6] European Commission (2017). Gender Equality 2017. Gender Equality, Stereotypes, and Women in Politics..

[7] Kohlberg L.A. (1966). A Cognitive—developmental Analysis of Children’s Sex Role Concepts and Attitudes.

[8] Bem S.L. (1981). Gender schema theory: A cognitive account of sex typing. Psychol. Rev..

[9] Lipowska M., Lipowski M., Pawlicka P. (2016). "Daughter and son: A completely different story”? Gender as a moderator of the relationship between sexism and parental attitudes. Health Psychol. Rep..

[10] Şalgam D. The Roles of Toys in Gender and Sexual Identity Construction in Early Childhood. Proceedings of the International Congress on Play and Toy.

[11] Liben L.S., Schroeder K.M., Borriello G.A., Weisgram E.S., Weisgram E.S., Dinella L.M. (2018). Cognitive consequences of gendered toy play. Gender Typing of Children’s Toys: How Early Play Experiences Impact Development.

[12] Blakemore J.E.O., Centers R.E. (2005). Characteristics of Boys’ and Girls’ Toys. Sex Roles.

[13] Miller C.L. (1987). Qualitative differences among gender-stereotyped toys: Implications for cognitive and social development in girls and boys. Sex Roles.

[14] Reich S.M., Black R.W., Foliaki T. (2018). Constructing Difference: Lego® Set Narratives Promote Stereotypic Gender Roles and Play. Sex Roles.

[15] Singer D., Golinkoff R.M., Hirsh-Pasek K. (2006). Play = Learning: How Play Motivates and Enhances Children’s Cognitive and Socialemotional Growth.

[16] Basow S. A., Levesque R. J. R. (2011). Gender Role and Identity. Encyclopedia of Adolescence.

[17] Doni E. (2021). Exposing Preschool Children to Counterstereotypical Professional Role Models Using Audiovisual Means: A Small Study in a Preschool in Greece. Early Child. Educ. J..

[18] Galotti K.M. (2011). Cognitive Development: Infancy Through Adolescence.

[19] Kollmayer M., Schultes M.-T., Schober B., Hodosi T., Spiel C. (2018). Parents’ Judgments about the Desirability of Toys for Their Children: Associations with Gender Role Attitudes, Gender-typing of Toys, and Demographics. Sex Roles.

[20] Campenni C.E. (1999). Gender Stereotyping of Children’s Toys: A Comparison of Parents and Nonparents. Sex Roles.

[21] Eisenberg N., Wolchik S.A., Hernandez R., Pasternack J.f. (1985). Parental Socialization of Young Children’s Play: A Short-Term Longitudinal Study. Child Dev..

[22] Yeung S.P., Wong W.I. (2018). Gender Labels on Gender-Neutral Colors: Do they Affect Children’s Color Preferences and Play Performance?. Sex Roles.

[23] Lipowska M., Łada-Maśko A.B., Lipowski M. (2021). Fathers’ Coping Strategies and Children’s Temperament as Predictors of Parental Attitudes among Polish Fathers. Men Masc..

[24] Karolewski I.P., Benedikter R. (2017). Poland’s conservative turn and the role of the European Union. Eur. Political Sci..

[25] Central Statistical Office (2021). Population. Size and Structure and Vital Statistics in Poland by Territorial Division in 2020.

[26] Ambroziak Ł., Dębkowska K., Gniadek J., Gniazdowski M., Jarząbek A., Kłosiewicz-Górecka U., Kukołowicz P., Rybacki J., Szpor A., Święcicki I. (2020). PIE: Wydatki świąteczne zdeterminują kształt PKB w IV kw. 2020 r. [Christmas spending will determine the shape of GDP in Q4 2020.]. Tyg. Gospod..

[27] Warat M., Krzaklewska E., Ratecka A., Slany K. (2017). Gender Equality and Quality of Life.

[28] Gruber S., Szołtysek M. (2016). The patriarchy index: A comparative study of power relations across historical Europe. Hist. Fam..

[29] Trybunał Konstytucyjny (2020). Planowanie Rodziny, Ochrona Płodu Ludzkiego i Warunki Dopuszczalności Przerywania Ciąży [Family Planning, Protection of the Human Fetus and Conditions for the Admissibility of Termination of Pregnancy]. https://trybunal.gov.pl/postepowanie-i-orzeczenia/wyroki/art/11300-planowanie-rodziny-ochrona-plodu-ludzkiego-i-warunki-dopuszczalnosci-przerywania-ciazy?fbclid=IwAR1LtvhsoCqTWHM_ZlIR_u3RPdZ3Q8VNwMr8OV34YOl2T0hhUQ-0YnPBN04.

[30] Walker S. Poland Abortion Ban Sets Stage for Women’s Strike Showdown. https://www.theguardian.com/world/2021/jan/28/poland-abortion-ban-sets-stage-for-womens-strike-showdown.

[31] Fine J.B., Mayall K., Sepúlveda L. (2017). The Role of International Human Rights Norms in the Liberalization of Abortion Laws Globally. Health Hum. Rights.

[32] Romaniuk J.R., Kotlarska-Michalska A., Farkas K.J. (2021). American perspectives on suicidality among men in Poland. Soc. Reg..

[33] Ritchie H., Roser M., Ortiz-Ospina E., Suicide Published Online at OurWorldInData.org. https://ourworldindata.org/suicide.

[34] Francis B. (2010). Gender, toys and learning. Oxf. Rev. Educ..

[35] Hartley B.L., Sutton R.M. (2013). A Stereotype Threat Account of Boys’ Academic Underachievement. Child Dev..

[36] Weaver-Hightower M. (2003). The “Boy Turn” in Research on Gender and Education. Rev. Educ. Res..

[37] Pansu P., Régner I., Max S., Colé P., Nezlek J.B., Huguet P. (2016). A burden for the boys: Evidence of stereotype threat in boys’ reading performance. J. Exp. Soc. Psychol..

[38] Régner I., Smeding A., Gimmig D., Thinus-Blanc C., Monteil J.-M., Huguet P. (2010). Individual Differences in Working Memory Moderate Stereotype-Threat Effects. Psychol. Sci..

[39] Sanson A., Muccio C.D. (1993). The influence of aggressive and neutral cartoons and toys on the behaviour of preschool children. Aust. Psychol..

[40] Hausmann R., Tyson L.D., Zahidi S. (2010). The Global Gender Gap Report 2010.

[41] Lewis M., Lockheed M. Social Exclusion and the Gender Gap in Education, Policy Research Working Paper No. (2008). 4562.

[42] Jirout J.J., Newcombe N.S. (2015). Building Blocks for Developing Spatial Skills: Evidence from a Large, Representative U.S. Sample. Psychol. Sci..

[43] Wieners B. Lego Is for Girls. https://www.bloomberg.com/news/articles/2011-12-14/lego-is-for-girls.

[44] Mattel About Barbie. https://barbie.mattel.com/en-us/about.html?icid=all_header_top-nav_about_p1.

[45] Dittmar H., Halliwell E., Ive S. (2006). Does Barbie make girls want to be thin? The effect of experimental exposure to images of dolls on the body image of 5- to 8-year-old girls. Dev. Psychol..

[46] Smith R., Nadin S., Jones S. (2019). Beyond the dolls house?. Qual. Mark. Res..

[47] Mattel The Dream Gap Project. https://barbie.mattel.com/en-us/about/dream-gap.html.

[48] Brabaw K. Mattel Is Launching Astrophysicist Barbie This Year (an Astronaut, Too!). https://www.space.com/astrophysicist-barbie-astronaut-doll-mattel-2019.html.

[49] Gould E., Schieder J. (2016). ‘Women’s Work’ and the Gender Pay Gap: How Discrimination, Societal Norms, and Other Forces Affect Women’s Occupational Choices-and Their Pay”. https://www.epi.org/publication/womens-work-and-the-gender-pay-gap-how-discrimination-societal-norms-and-other-forces-affect-womens-occupational-choices-and-their-pay/#epi-toc-9.

[50] Manea C.-N. (2013). The Influence of Hometown Size on the Development of Gender Stereotypes in Children. Procedia Soc. Behav. Sci..

